# Temperature-Dependent Twist of Double-Stranded RNA Probed by Magnetic Tweezer Experiments and Molecular Dynamics Simulations

**DOI:** 10.1021/acs.jpcb.3c06280

**Published:** 2024-01-10

**Authors:** Hana Dohnalová, Mona Seifert, Eva Matoušková, Misha Klein, Flávia S. Papini, Jan Lipfert, David Dulin, Filip Lankaš

**Affiliations:** † Department of Informatics and Chemistry, University of Chemistry and Technology Prague, Technická 5, 166 28 Praha 6, Czech Republic; ‡ Junior Research Group 2, Interdisciplinary Center for Clinical Research, Friedrich-Alexander-University Erlangen-Nürnberg, Cauerstr. 3, Erlangen 91058, Germany; § Department of Physics and Astronomy and LaserLaB Amsterdam, Vrije Universiteit Amsterdam, De Boelelaan 1081, Amsterdam 1081 HV, The Netherlands; ∥ Soft Condensed Matter and Biophysics, Department of Physics and Debye Institute, Utrecht University, Utrecht 3584 CC, The Netherlands

## Abstract

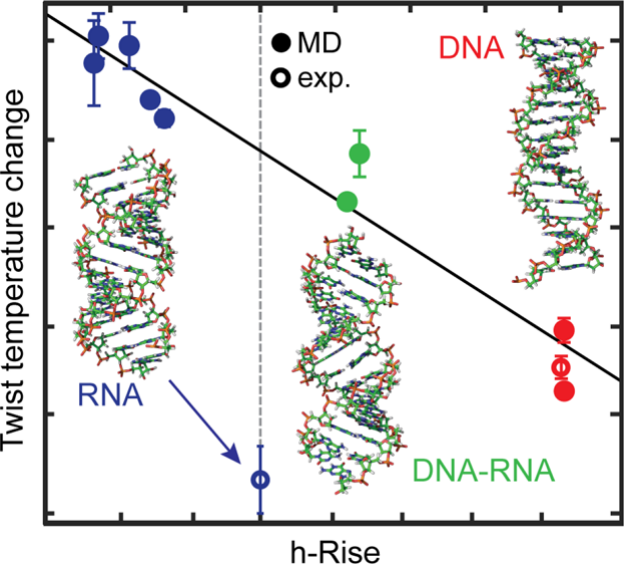

RNA plays critical roles in the transmission and regulation of genetic information and is increasingly used in biomedical and biotechnological applications. Functional RNAs contain extended double-stranded regions, and the structure of double-stranded RNA (dsRNA) has been revealed at high resolution. However, the dependence of the properties of the RNA double helix on environmental effects, notably temperature, is still poorly understood. Here, we use single-molecule magnetic tweezer measurements to determine the dependence of the dsRNA twist on temperature. We find that dsRNA unwinds with increasing temperature, even more than DNA, with Δ*T*_wRNA_ = −14.4 ± 0.7°/(°C·kbp), compared to Δ*T*_wDNA_ = −11.0 ± 1.2°/(°C·kbp). All-atom molecular dynamics (MD) simulations using a range of nucleic acid force fields, ion parameters, and water models correctly predict that dsRNA unwinds with rising temperature but significantly underestimate the magnitude of the effect. These MD data, together with additional MD simulations involving DNA and DNA–RNA hybrid duplexes, reveal a linear correlation between the twist temperature decrease and the helical rise, in line with DNA but at variance with RNA experimental data. We speculate that this discrepancy might be caused by some unknown bias in the RNA force fields tested or by as yet undiscovered transient alternative structures in the RNA duplex. Our results provide a baseline to model more complex RNA assemblies and to test and develop new parametrizations for RNA simulations. They may also inspire physical models of the temperature-dependent dsRNA structure.

## Introduction

Nucleic acid double helices in their DNA and RNA forms play fundamental roles in biology. DNA is a carrier of genetic information in all cellular life. RNA performs critical functions in the transmission of genetic information and can adopt a variety of structures, the double helix being a prominent structural motif.^1^ Double-stranded RNA (dsRNA) also serves as either the genome or a replication intermediate for many RNA viruses.^2^ Long dsRNA occurs in cells and signals cellular dysfunction, such as dsRNA originating from long Alu retroelements in the absence of deamination by ADAR1^3^ or from malfunctioning mitochondria.^4^ In addition to their biological role, DNA and RNA duplexes are employed as basic building blocks of artificial nanostructures.^5^

Life can flourish in a range of temperatures: extreme thermophiles can tolerate 100 °C, while extreme psychrophiles can survive at nearly 0 °C.^6^ To thrive in a broad temperature range, organisms need to use efficient strategies of thermal adaptation. Mechanisms of thermal adaptation at the molecular level in general and thermal effects on nucleic acid properties and function in particular are now starting to be understood.^7,8^ For instance, temperature has been found to play a critical role in defining the outcome of viral infections and the direction of evolution of RNA viruses.^8^ Temperature-dependent properties of DNA and RNA double helices also play a role in nucleic acid nanostructures, which can operate in a broad temperature range and may even be thermally activated to perform functions related to biochemical diagnostics or drug delivery.^9^ Thus, we need to better understand how the structure of nucleic acid double helices in their DNA and RNA forms depends on temperature.

A number of studies have focused on temperature-dependent properties of double-stranded (ds) DNA. Thermal effects on the dsDNA bending persistence length^10^ and twist stiffness^11^ have been examined using a variety of methods. Based on several lines of evidence, a two-state model of dsDNA structure and stiffness, including effects of temperature and other factors, has been formulated.^12^ Physical models informed by atomic-resolution molecular dynamics (MD) simulations were used to probe temperature effects on dsDNA structure and elasticity.^13^ In previous work, we have determined changes of dsDNA twist with temperature by magnetic tweezer (MT) measurements and quantitatively compared the experimental findings to atomic-resolution and coarse-grained MD simulations.^14^ A follow-up MD study focused on temperature-dependent dsDNA bending and elongation.^15^ A similar methodology combining MT measurements and MD simulations was used to examine the dsDNA twist dependence on the ionic environment.^16^ Quantitative agreement between MT measurements and all-atom MD data, at least in parts, suggests that MD simulations of DNA oligomers ∼3 helical turns long, represented at atomic resolution and at the microsecond timescale, may offer a powerful and quantitative approach to probe thermal and ionic effects on DNA structure. While these works elucidated many aspects of the temperature-dependent shape and stiffness of DNA duplexes, how the structural properties of RNA double helices are affected by temperature remains largely unknown.

In this work, we examined the temperature dependence of the twist of RNA and DNA double helices. We used magnetic tweezers (MT) to measure the temperature-dependent twist of dsRNA, and we found that dsRNA twist decreases with temperature. The slope inferred from the experiment, −14.3 ± 0.7°/(°C·kbp), is higher than the one for dsDNA, −11.0 ± 1.2°/(°C·kbp), previously reported using the same experimental approach.^14^

We complemented the experiments with atomic-resolution MD simulations of double-stranded RNA and DNA. To better understand the microscopic mechanism of twist temperature dependence, we also performed MD simulations of a DNA–RNA hybrid duplex. We simulated 33 base-pair (bp) oligomers, systematically testing several parametrizations of interatomic interactions (force fields) for the nucleic acid, water, and ions. To get more insight into the sequence dependence of thermal effects on RNA twist, we also simulated another 25 bp dsRNA oligomer with a different base composition.

The dsDNA MD simulations yield a decrease of twist with an increase in temperature in quantitative agreement with the MT experiment. The dsRNA simulations also indicate a decrease of dsRNA twist with temperature, agreeing qualitatively with the MT measurement. However, the simulated magnitude of the change dramatically deviates from the MT experiment; the dsRNA twist decrease inferred from MD is at least 3 times lower than the MT value. The two simulated RNA oligomers exhibited a similar twist change with temperature, despite their very different base compositions, adding confidence to the generality of the MD results. The dsRNA twist decrease observed in MD is moderately reduced upon increasing the salt concentration from 150 mM to 1 M KCl. Furthermore, the MD data suggest that the twist–temperature slopes of the dsRNA, dsDNA, or hybrid oligomer are tightly correlated with the oligomer compaction quantified by its helical rise. While this dependence is in line with experimental data for dsDNA, it again disagrees with dsRNA experimental values. We speculate that the discrepancy between experiment and simulation for the RNA duplex may be caused by some systematic bias in RNA force fields or by the very different length scales and timescales probed in the MT experiment and in the MD simulations. The latter possibility would suggest the existence of as yet undisclosed transient structures within the RNA duplex, which, contrary to known transient structures in dsDNA, would make the dsRNA twist thermal response length scale- and timescale-dependent.

## Materials and Methods

### Magnetic Tweezer Instrument Measurement of dsRNA Twist

The detailed description of the magnetic tweezer instrument is provided in ref (17). A pair of permanent magnets (neodymium 5 mm cubes, W-05-G, SuperMagnete, Switzerland) were mounted above a flow chamber, which itself was mounted on a custom inverted microscope. The magnets were vertically mounted with a 1 mm gap separating them,^18^ and their vertical position and rotation were controlled by two linear motors, i.e., M-126.PD1 and CD-150, respectively (Physik Instrumente, Germany). The flow chamber was illuminated by a collimated LED (660 nm, 400 mW, LH CP7P, Hechigen, Germany; spherical condenser, NA = 0.79, Thorlabs, Germany) and imaged by a 50× oil immersion objective (CFI Plan Achro 50 XH, NA 0.9, Nikon, Germany), whose vertical position was adjusted using a high-resolution piezo stage (P-726 PIFOC and E-753 piezo controller, Physik Instrumente, Germany). The image was projected onto a CMOS camera (Dalsa Falcon2 FA-80-12M1H, Stemmer Imaging, Germany) by a 200 mm focal length achromatic doublet (Thorlabs, Germany). The temperature in the field of view was controlled using a resistive foil heater with an integrated 10 MΩ thermistor (HT10K, Thorlabs) wrapped around the objective and a PID temperature controller (TC200 PID), as described in ref (19).

### Fabrication of the dsRNA Construct

The coilable dsRNA construct fabrication is described in detail in ref (20). Plasmid DNA pBB10 was used as a template for PCR with primers containing the T7 promoter, and the purified PCR products were subsequently used as a template to produce RNA *in vitro* using a RiboMAX large-scale RNA production system, T7 (Promega GmbH, Mannheim, Germany). The biotinylated and digoxigenin-labeled handles were produced by adding biotin-UTP and digoxigenin-UTP, respectively, into the *in vitro* transcription reaction solution.^20^ The RNAs were purified with an RNeasy MinElute kit (Qiagen), and concentrations were determined using a Nanodrop. The 5′-ends of the RNA were digested to have only one phosphate moiety.^20^ The final dsRNA construct was assembled by annealing four single-stranded RNA strands together: an ∼4 kb-long ssRNA to which three ssRNAs anneal, i.e., the biotin and the digoxigenin handles, and an ∼3.3 kb-long RNA. It was subsequently ligated with T4 RNA ligase 2 (NEB).^20^ The dsRNA sequence used in the MT experiment is listed in Supplementary Methods.

### Preparation of the Flow Chamber

Description of the flow chamber assembly and preparation can be found in ref (21). Shortly, the flow chamber was made of a double layer of Parafilm (Parafilm M, P7793, Sigma-Aldrich, Germany) sandwiched by two #1 coverslips (24 mm × 60 mm, Menzel GmbH, Germany), with a channel carved in the parafilm. The top coverslip had two ∼1 mm-diameter holes drilled at both ends of the long side using a sandblaster (Vaniman, USA). The two holes acted as the inlet and outlet for the flow chamber. Both top and bottom coverslips were thoroughly washed by sonication for 30 min in a 2% (V/V) Hellmanex III solution in demineralized water, rinsed with demineralized water, and dried. The bottom coverslip was coated with an ∼0.1% m/V nitrocellulose solution in amylacetate. The flow chamber was sealed by melting the parafilm for ∼30 s at ∼90 °C. After mounting the flow chamber on the magnetic tweezer setup, 1 μm polystyrene reference beads were flushed in 1/1000 dilution in phosphate-buffered saline (PBS), LB11 (Sigma-Aldrich, Germany) and incubated until a few of them attached to the nitrocellulose-coated bottom coverslips, and the excess was flushed away with ∼1 mL of PBS. Antidigoxigenin antibodies (50 μg/mL in PBS, Roche, Switzerland) were flushed in the flow chamber, incubated for 30 min, and then rinsed with 1 mL of high-salt TE buffer (10 mM Tris, 1 mM EDTA pH 8.0, and 2 mM sodium azide, supplemented with 700 mM NaCl). After ∼10 min of incubation of the high-salt buffer, the flow chamber was rinsed with TE 1× buffer (10 mM Tris, 1 mM EDTA pH 8.0, and 2 mM sodium azide, supplemented with 150 mM NaCl). Bovine serum albumin (10 mg/mL in PBS, lyophilized stock from Sigma-Aldrich) was then flushed in the flow chamber, incubated for 30 min, and subsequently flushed out with 1 mL of TE 1× buffer. Ten μL of Dynabeads MyOne Streptavidin T1 magnetic beads (Thermo Fisher, Germany, cat. no. 65604D) were washed twice in TE 1× buffer and subsequently mixed with ∼0.2 ng of coilable ∼3.3 kbp dsRNA and 1 mg/mL bovine serum albumin (BSA, New England Biolabs). The dsRNA-attached beads were then washed once to remove the excess RNA, resuspended in 40 μL of TE 1× buffer, and flushed in the flow cell. Following ∼10 min of incubation, the excess magnetic beads were flushed out with 1 mL of TE 1× buffer followed by flushing in 1 mL of phosphate-buffered saline (PBS), in which the experiments took place.

### Magnetic Tweezer Experiments

To determine whether the tethers were coilable, a test rotation–extension experiment was performed, rotating the magnets from −20 turns to +20 turns at 0.4 turns/s and applying a force of 4 pN.^22^ A coilable molecule then showed no significant change in extension in negative supercoils, while its end-to-end extension decreased in positive supercoils.^20,23^ A noncoilable molecule showed no change in extension in both positive and negative supercoils, while a bead attached via multiple dsRNA tethers showed a decrease in extension upon applying both positive and negative turns.

**Figure 1:**
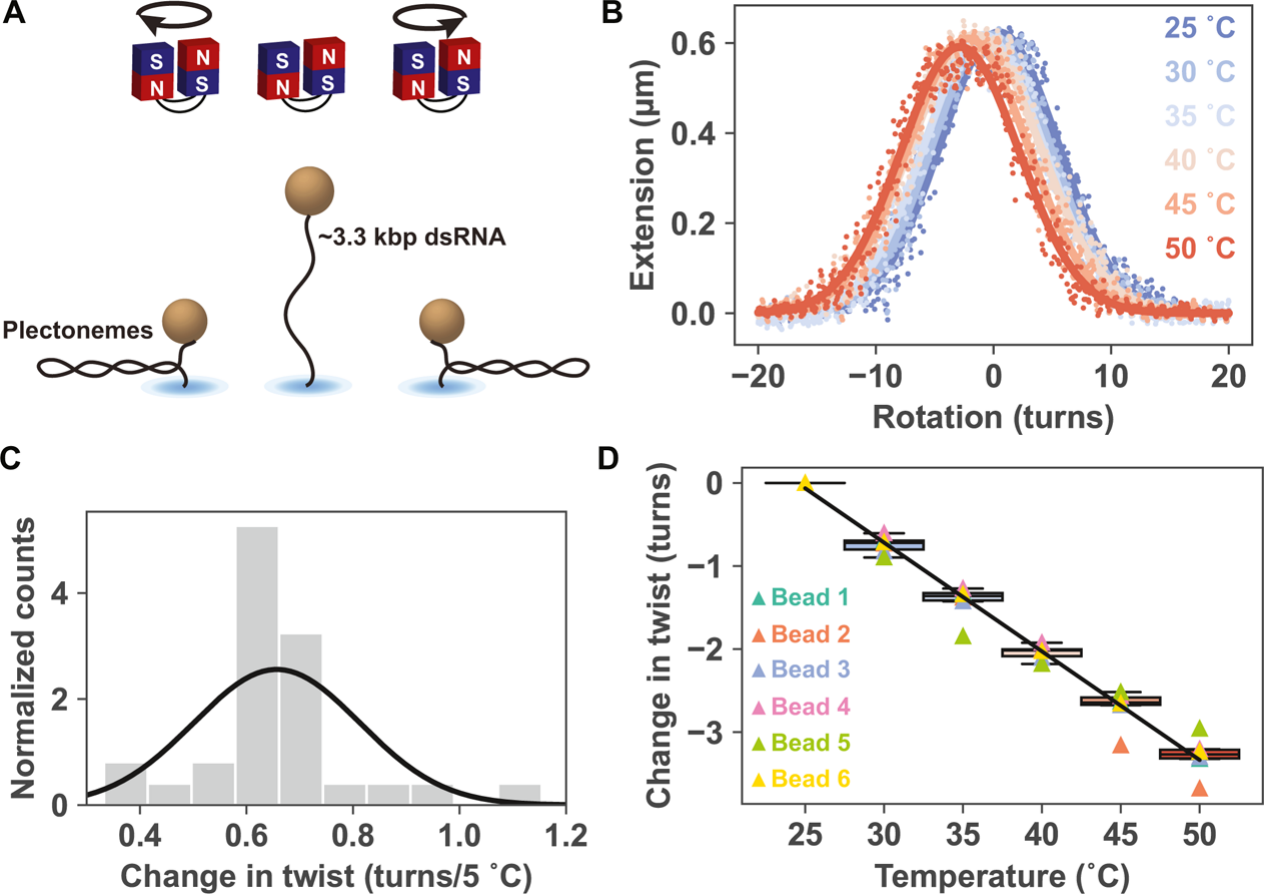
Single-molecule magnetic tweezer experiments reveal that dsRNA twist decreases with increasing temperature. (A) Schematic of the rotation–extension experiment performed with a magnetic tweezer instrument. The magnets are rotated from negative (left) to positive (right) turns, showing the formation of plectonemes upon over- and underwinding of the RNA. (B) Rotation–extension traces were performed at various temperatures (indicated in the legend) for a single coilable dsRNA tether. The dots represent the 10 times decimated data, and the solid lines are their respective Gaussian fits. (C) Distribution of the difference in turns at the maximum tether extension, i.e., the center of the rotation–extension curve, upon decreasing the temperature by 5 °C, extracted from Gaussian fits to extension vs rotation data for consecutive temperatures, for *N* = 6 independent dsRNA tethers. The solid line is a Gaussian fit with mean ± 2 × SEM of (0.65 ± 0.06) turns, corresponding to Δ*T*_wRNA_ = (−14.3 ± 1.2)°/(°C·kbp). (D) Position of the maximum tether extension as a function of temperature, with respect to the value at 25 °C. The triangles are measurements for *N* = 6 independent dsRNA tethers; the box plot represents the distribution across the beads for each temperature. At 25 °C, the twist is 0° by definition, so that the box reduces to a line. Using temperature as the regressor for the twist change with respect to 25 °C results in the straight fitting line shown. Its slope yields Δ*T*_wRNA_ = (−14.3 ± 0.7)°/(°C·kbp).

To extract the dsRNA twist dependence on temperature, we performed dynamic rotation–extension experiments of the dsRNA in PBS at an ∼0.3 pN force (Figure 1A). At such low forces, the rotation–extension of a coilable dsRNA tether is symmetric, with an approximately Gaussian shape, where the maximum extension corresponds to the torsionally relaxed molecule.^20,23^ The data were recorded at a 58 Hz camera acquisition frequency, and the magnets were rotated at 0.4 turns/s from −20 to +20 turns. Dynamic rotation–extension experiments were repeated at each temperature, from 25 to 50 °C with incremental steps of 5 °C, as previously described for coilable dsDNA^19^ (Figure 1B). The rotation–extension curves were averaged 10 times and subsequently fitted with a Gaussian function using a least-squares fitting routine (Figure 1B). The number of turns at maximum extension was extracted for each dsRNA tether from the Gaussian fit peak position, represented as a function of temperature with the value at 25 °C set to zero (Figure 1B,C), and (“simple”) linear regression was used to fit a line through these data points. Performing linear regression enables the estimation of the slope (Δ*T*_w_) as well as the standard deviation of the estimated slope. The reported error is twice this standard deviation, representing an ∼95% confidence interval. From this fit, the dependence of the dsRNA twist changes on the temperature was extracted (Figure 1D).

### Molecular Dynamics Simulations and Analysis

We simulated the 33 bp oligomer used in a previous study,^14^ whose sequence of the reference strand reads GAGAT GCTAA CCCTG ATCGC TGATT CCTTG GAC. The RNA version of the duplex has a sequence where U replaces T in both strands, and the hybrid sequence was obtained by replacing T by U in the complementary strand only. We also simulated a 25 bp RNA oligomer of the sequence CGACU CUACG GAAGG GCAUC UGCGC employed in earlier works.^24,25^

We performed unrestrained atomic-resolution MD simulations with explicitly represented water molecules and ions using the Amber17 suite of programs. Each system was simulated at 7, 17, 27, 37, and 47 °C. A DNA simulation using the bsc1 force field^26^ was produced, complementing the OL15^27^ simulation reported previously,^14^ while the χOL3^28^ and Shaw^29^ force fields were used for RNA. The Dang^30^ and Joung–Cheatham (JC)^31^ ion parameters and the SPC/E as well as TIP4PEw water models were utilized, with the exception of the Shaw RNA force field, which was combined with its recommended CHARMM22 ion parameters^32^ and the TIP4P-D water model.^33^ In addition to the standard physiological concentration of 150 mM KCl, we also investigated dsRNA under high-salt conditions of 1 M KCl. In addition, dsRNA simulations at 150 mM NaCl were performed to test the dependence of the results on the ion type. The parameter combinations utilized are shown in Table S1.

An additional series of MD data was produced using the TIP3P water model,^34^ combined with the JC ion parameters as commonly done.^35,36^ The simulations at 7 to 47 °C (see above) were complemented by another series of otherwise identically produced MD at 20 to 40 °C with a step of 5 °C, to better understand the behavior of the simulated systems at near-ambient temperatures. The simulations are listed in Table S2.

**Figure 2:**
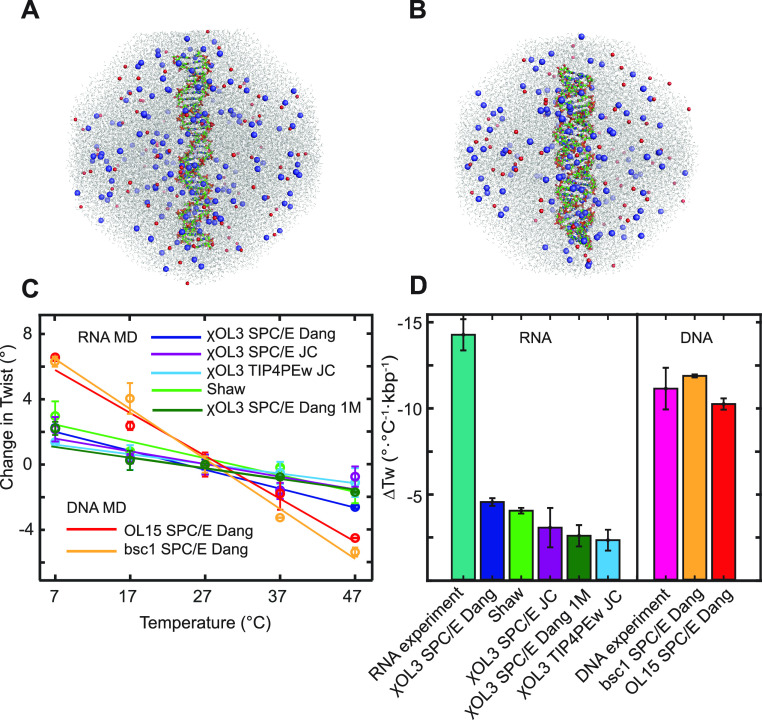
Molecular dynamics (MD) simulations of temperature-dependent double-stranded RNA and DNA twist compared to magnetic tweezer (MT) measurements. (A,B) The simulated systems containing 33 base-pair dsDNA (A) and dsRNA (B), together with the K^+^ cations (blue), Cl^–^ anions (red), and water molecules (gray), are each immersed in an octahedral simulation box. The system sizes are shown approximately to scale: the dsRNA system is smaller since the RNA duplex is shorter than the DNA one containing the same number of base pairs. (C) The simulated temperature changes of the end-to-end twist are well-approximated by least-squares linear fits, and all indicate a decrease of twist with rising temperature. (D) Comparison of the simulated twist–temperature slopes to magnetic tweezer measurements. While the MD data quantitatively agree with the dsDNA experiment, the MD simulations largely underestimate the measured twist decrease of the RNA duplex. The dsDNA MT experimental value is taken from ref (14). The MD data shown are for the 33 bp oligomer. The values with errors, as well as those for the other, 25 bp dsRNA, and for the other twist definitions and ionic conditions are in Table S1.

The DNA and RNA duplexes were built in their canonical B- and A-forms, respectively, using the *nab* module of Amber. To build the hybrid, the 3D-NuS server^37^ with the *sequence-specific model* and *nmr* options was used. The systems were immersed in an octahedral periodic box containing the duplex, water molecules, K^+^/Na^+^ ions to neutralize the duplex charge, and additional K^+^/Na^+^ and Cl^–^ ions to mimic the desired concentration of 150 mM KCl or NaCl or 1 M KCl (Figure 2A,B). They were then subjected to a series of energy minimizations and short MD runs before starting the production of MD trajectories, 1 μs each. For the 150 mM systems, the standard Amber hydrogen mass repartitioning, the time step of 4 fs, and a 9 Å nonbonded cutoff were employed. The 1 M systems were unstable in such conditions, so that a 2 fs time step, no mass repartitioning, and a 10 Å cutoff were used. Snapshots were taken every 10 ps. The oligomer global twist was measured as the end-to-end or mean plane twist between reference frames at the oligomer ends. The end frames were obtained by projecting the end base-pair frames (defined as in the 3DNA algorithm^38^) onto the local helical axis, computed by averaging the axes of the two steps containing the pair. The helical axes of the steps were calculated as in 3DNA. The end-to-end twist between the end frames was then defined exactly as the local twist between the neighboring base-pair frames in 3DNA. The changes of the end-to-end twist defined in this way are invariant with respect to the constant offset rotation of the end frames around their *z*-axes.^14^ We also probed the global twist defined by the sum of helical twists of the base-pair steps within the analyzed fragment, either computed as in 3DNA or extracted from the output of the Curves+ conformational analysis program.^39^ Details of the protocol can be found in the previous work.^14^ Only the inner 27 bp of the 33 bp sequence and the inner 19 bp of the 25 bp sequence were analyzed, and 3 bp at each end were excluded. In addition to the data from the whole trajectories, we also examined filtered data, where only snapshots with intact hydrogen bonds in the analyzed part were taken into account. A hydrogen bond was considered to be present if the distance between heavy atoms was less than 4 Å. The errors were estimated as the mean absolute difference between the value for the whole trajectory and the value for each of its halves.

## Results

### Magnetic Tweezer Measurements Reveal a Strong Decrease of Double-Stranded RNA Twist with Increasing Temperature

We used a temperature-controlled high-throughput magnetic tweezer (MT) setup^19^ (Materials and Methods), where an ∼3.3 kbp dsRNA molecule tethered a 1 μm-diameter magnetic bead to the flow cell surface (Figure 1A). Nick-free and end-labeled dsRNA constructs were generated by annealing single-stranded RNA followed by ligation (Materials and Methods).^20^ The dsRNA molecule is flanked by two handles, one randomly biotin-labeled at multiple points to attach to the magnetic bead and the other randomly digoxygenin-labeled at multiple sites to bind to the antidigoxygenin that functionalized the flow chamber glass surface. Permanent magnets were mounted above the flow cell, and their height was adjusted to apply precisely calibrated stretching forces on the nucleic acid tether.^17,18,40^ Rotation of the magnets enabled precise control of the tethered molecule supercoiling density. We have previously shown that dsRNA exhibits an overall extension vs applied rotation response very similar to dsDNA.^23^ The dsRNA molecules were tested to determine their coilability at the start of the experiment, as described in Materials and Methods. We then measured extension vs applied rotation curves at a force of 0.3 pN, which is much lower than the stretch moduli of dsRNA (350–500 pN).^23,41^ In this low force regime, the extension vs rotation response is symmetric, i.e., the dsRNA molecule forms plectonemes for both negative and positive supercoiling, enabling Gaussian fit analysis of each rotation–extension (Figure 1A,B and Figure S1A). We found that the maximum extension in the rotation–extension curve systematically shifts to lower turns with increasing temperature, e.g., from 0.5 turns at 25 °C to −2.8 turns at 50 °C (Figure 1B) and generally by −3.3 turns at 50 °C with respect to 25 °C measurement (Figure S1B). This systematic shift with temperature indicates that dsRNA unwinds when heated, a result qualitatively similar to DNA. We determined a change in the twist for dsRNA to be Δ*T*_w__RNA_ = (−14.3 ± 0.7)°/(°C·kbp) (Figure 1C,D), i.e., dsRNA unwinds more than dsDNA since Δ*T*_wDNA_ = (−11.0 ± 1.2)°/(°C·kbp).^14,19^ Our value for Δ*T*_w__RNA_ is in excellent quantitative agreement with an independent measurement published recently by Tian et al.,^25^ also using magnetic tweezers but a different dsRNA sequence and a different but overlapping temperature range (20–35 °C, compared to 25–50 °C in this work), which reported Δ*T*_w__RNA_ = 15 ± 2°/(°C·kbp) at the same ionic strength as our measurements and no dependence of the twist change with temperature on KCl concentration in the range of 0.05–1 M, within experimental error.

### Extension of Torsionally Relaxed dsRNA Drops at High Temperature

The maximum extension remained largely constant at all temperatures but at 50 °C, where it decreased by approximately 5% (0.61 μm at 50 °C vs 0.64 μm at the other temperatures) (Figure S1B). The width that we extracted from the Gaussian fits remained constant with respect to temperature (Figure S1B). Furthermore, the local slope of the rotation–extension curves, when adjusted for their respective lateral shifts, overlaps, demonstrating that the rotation–extension curves remain symmetric and do not significantly broaden in the investigated temperature range (Figure S1C), which suggests that the RNA remained double-stranded.

To get insight into possible mechanisms of the observed extension drop at 50 °C, we assume that the torsionally relaxed RNA molecule is well-described by an inextensible wormlike chain (WLC) model. This assumption is consistent with experimental data for kilobase-long dsDNA^42,43^ as well as dsRNA^23,41,44^ at ambient temperatures and at forces where enthalpic stretching is negligible. This is the case in our experiment where dsRNA is pulled by a force *f* = 0.3 pN, 3 orders of magnitude lower than the dsRNA stretch modulus of 350–500 pN.^23,41^ In this regime, the resistance to pulling is purely entropic and is dictated by the molecule’s bending persistence length *P*. It has been shown in ref (43) that, for forces satisfying the condition *fP* ≫ *k*_B_*T*, the ratio of the WLC extension *z* and its contour length *L* obeys the approximate relation *z*/*L* = 1 – (*k*_B_*T*/4*fP*)^1/2^. Ample experimental evidence^23,41,44^ suggests that the persistence length of dsRNA at ambient temperature and physiological salt concentration is around 60 nm. Assuming *P* = 60 nm and *T* = 300 K, we obtain *fP* = 18 pN nm, much greater than *k*_B_*T* = 4.14 pN. nm, validating the approximation. The persistence length *P* of a WLC is related to its bending stiffness *A*_b_, a material property of the chain, as *P* = *A*_b_/*k*_B_*T*, so that *P* = 60 nm at *T* = 300 K implies *A*_b_ = 248 pN nm^2^. If *A*_b_ is temperature-independent, then *P* decreases with temperature. Since we are interested in temperature dependence of dsRNA material properties, it will be more convenient to work with *A*_b_ rather than *P*. The force–extension relation then takes the form
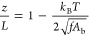
1

This relation suggests that the extension drop from *z*_1_ = 0.64 nm at *T*_1_ = 318 K (or 45 °C) to *z*_2_ = 0.61 nm at *T*_2_ = 323 K (or 50 °C) at constant pulling force *f* may be achieved through various mechanisms involving the contour length *L* and the bending rigidity *A*_b_. Two limiting cases are easy to compute. If the bending rigidity is temperature-independent, then the observed extension drop is consistent with shortening the contour length by 5%. If, by contrast, the contour length does not depend on temperature, then the diminished extension implies a decrease of the dsRNA bending rigidity from 248 pN nm^2^ down to 198 pN nm^2^, or by 20%. The shortening of the contour length can in principle be achieved by the formation of extrahelical structures; the softening might be a consequence of transient alternative structures within the helix. We stress that whatever mechanism may lead to the decreased dsRNA extension at 50 °C, it does not affect the thermally induced change of dsRNA twist, as the twist decreases linearly in the whole temperature range of 25–50 °C (Figure 1D).

### Microsecond-Scale MD Simulations Indicate a Smaller Decrease of dsRNA Twist with Temperature than the MT Experiments

To understand the microscopic origin of the observed changes in the twist of the dsRNA double helix with temperature and to quantitatively test available force fields for nucleic acids, we turned to all-atom molecular dynamics (MD) simulations. We performed microsecond-long MD simulations of a 33 bp dsRNA sequence, testing different force fields for the nucleic acid, water, and ions. In addition to the ion concentration of 150 mM KCl, we also examined dsRNA at 150 mM NaCl and at high-salt conditions of 1 M KCl. For comparison, we ran analogous simulations with 150 mM KCl for the dsDNA version of the same oligomer using the bsc1 force field for DNA, complementing the previously reported simulation^14^ using the OL15 force field. To provide insight into the sequence specificity of the temperature-dependent dsRNA twist, we simulated another shorter (25 bp) dsRNA oligomer with a different base composition. The GC content of the inner 27 bp included in the analysis for the longer duplex is 48%, compared to 58% GC for the analyzed inner 19 bp of the shorter duplex. Examples of the simulated systems are visualized in Figure 2A,B, and the DNA and RNA force field combinations employed (Materials and Methods) are listed in Table S1.

Temperature changes of the end-to-end twist obtained from MD for the 33 bp oligomer, together with the twist changes deduced from the MT experiments, are shown in Figure 2C,D, and the numerical values with errors are in Table S1. The end-to-end twist for all the simulations decreases with temperature, the dependence being close to linear (Figure 2C). The fitted MD temperature slopes and experimental MT values are shown in Figure 2D. The end-to-end twist decreases with increasing temperature for dsDNA obtained using OL15 and bsc1 force fields are both within the error margins of the MT experiment. Thus, quantitative agreement between the MT measurement and the OL15 simulations for DNA reported previously^14^ is extended in this work also to the case of the bsc1 force field.

For dsRNA, all MD simulations again predict a decrease of the end-to-end twist with rising temperature, in qualitative agreement with the MT experiments. However, the magnitude of the simulated decrease is significantly underestimated compared with the MT experimental value. Indeed, the simulations indicate a change between −4.5 ± 0.2 and −2.3 ± 0.6°/(°C·kbp) depending on the force filed and ionic conditions, smaller than the dsDNA value and more than 3 times smaller than the experimental MT results (Figure 2C,D and Table S1). Importantly, the 33 and 25 bp oligomers, when simulated using the same MD parametrization, yield twist changes identical within statistical error (Table S1). Replacing 150 mM KCl with 150 mM NaCl moderately reduces the dsRNA twist decrease with the temperature inferred from MD, and the twist decrease is further diminished in high-salt conditions of 1 M KCl (Figure 2C,D andTable S1).

The discrepancy between experiment and simulation common to all the force fields tested persists despite differences between the individual force field parametrizations. The χOL3 dsRNA force field, employed together with the SPC/E water model and 150 mM KCl using the Dang ion parameters, yields the highest negative slope, closely followed by the Shaw force field with its recommended TIP4P-D water and CHARMM22 ions (Figure 2C,D andTable S1). The two simulations using the Joung–Cheatham ion parameters give a somewhat lower temperature slope, while the effect of the water model (three-point or four-point) is minor (Figure 2C,D and Table S1).

### Simulated Twist–Temperature Slopes Tightly Correlate with Duplex Compaction

To gain further insight into the structural mechanism of the twist temperature change, we complemented our double-stranded RNA and DNA data by the MD simulations of a DNA–RNA hybrid. We again used a 33 bp oligomer of the same sequence as the DNA but with T replaced by U in the complementary strand (Materials and Methods). Two force field combinations were tested: the RNA strand was modeled using the χOL3 force field, while OL15 or bsc1 was used for the DNA strand. Even though there are currently no direct experimental data available for the thermally induced twist change of DNA–RNA hybrids, the inclusion of the hybrid oligomer enables us to investigate a mechanism of the twist temperature dependence common to all three simulated nucleic acid duplex variants.

**Figure 3:**
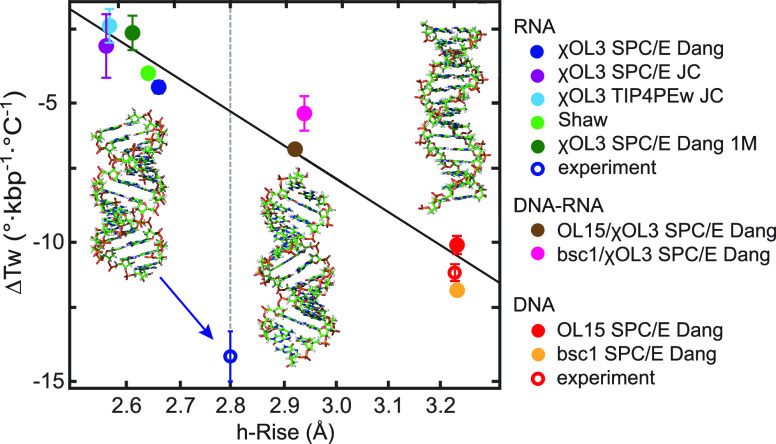
Temperature dependence of twist for double-stranded DNA, RNA, and hybrid DNA–RNA plotted against the helical rise. The dsDNA MT values from previous work^14^ together with the h-rise from ASAXS measurements^45^ (red empty circle), as well as the dsRNA MT value from this work and the consensus dsRNA h-rise^44^ (empty blue circle highlighted by the blue arrow), are shown as experimental data points. The MD data (end-to-end twist and mean helical rise) shown as solid circles follow a linear relationship and agree with the DNA experimental data. However, the MD simulations disagree with the dsRNA experimental data that exhibit a much more negative twist temperature change, even stronger than that for dsDNA. All of the RNA MD simulations somewhat underestimate the helical rise, producing more compact structures than expected. However, even if the h-rise is corrected to the consensus value (vertical broken line), the linear relationship would still yield the dsRNA twist–temperature slope much weaker than the MT experiment. Data for the 33 bp oligomers are shown. Fragments of the MD starting structures of dsDNA (right), the hybrid (middle), and dsRNA (left) are shown as well. Errors in MD h-rise values are very small and are omitted for clarity.

Figure 3 shows the twist–temperature slope inferred from MD as a function of the mean helical rise. Values for all the duplex variants (DNA, RNA, and hybrid), all force fields, and ionic conditions examined follow a clear trend: the smaller the helical rise, the weaker the twist temperature decrease. Moreover, the relationship is very close to linear (*R*^2^ = 0.98, straight line in Figure 3). A smaller helical rise means a shorter distance between base pairs measured along the helical axis, i.e., a shorter, more compact double helix. The MD simulations, therefore, suggest a mechanism of twist temperature dependence common to the DNA, RNA, and hybrid duplexes: the more compact the duplex is, the less sensitive its twist is to temperature changes.

dsDNA, with the largest helical rise, also exhibits the largest twist decrease (Figure 3, on the right). For the experimental dsDNA data point (Figure 3, red empty circle), we use a value of 3.23 Å for the rise, which is the distance between dsDNA base pairs in solution measured using anomalous small-angle X-ray scattering (ASAXS) with gold labels (3.23 ± 0.1 Å^45^), and the experimentally determined twist decrease from MT measurements.^14^ The dsDNA experimental and simulated data are close to each other, indicating that microsecond-scale MD simulations can quantitatively reproduce not only the experimental twist–temperature slope but also the measured DNA helical rise in solution.

The situation is very different for dsRNA (Figure 3, at the left). The experimental dsRNA data point in Figure 3 (blue empty circle, highlighted by an arrow) represents our MT value of −14.4 ± 0.7°/(°C·kbp), together with the dsRNA consensus helical rise in solution containing monovalent ions (2.8 ± 0.1 Å, see ref (44) and references therein). All dsRNA MD simulations yield twist changes with temperature much smaller than the MT experiment, and they also somewhat underestimate the RNA helical rise compared to its experimentally determined value in solution. However, even if the underestimated MD helical rise is corrected to the consensus value, the linear relationship would imply the slope of around −5°/(°C·kbp), as indicated by the intersection between the fitting line and the 2.8 Å vertical line (Figure 3). This is still a much weaker effect than the measured value. Increasing the salt concentration from 150 mM to 1 M KCl further decreases the simulated dsRNA rise, presumably due to stronger screening of the phosphate–phosphate electrostatic repulsion (Figure 3 and Table S1). The twist–temperature slope also somewhat decreases, in agreement with the linear relation in Figure 3. As for the simulated hybrid DNA–RNA duplex, its helical rise is slightly lower than but close to the experimental value of ∼3.0 Å,^46^ and the twist–temperature slope is around −6°/(°C·kbp), both lying between the MD values for dsDNA and dsRNA.

Thus, while the linear model agrees quantitatively with experimental data for dsDNA, it quantitatively deviates from the dsRNA experimental data that indicate a much stronger twist decrease with temperature, even if the model is corrected for the underestimated dsRNA helical rise.

The atomic-resolution information obtained from MD simulations enables us to get further insight into the microscopic mechanism of the temperature-induced twist change observed in MD of dsDNA, dsRNA, and hybrid duplexes. In particular, the twist change might, in principle, be caused by the formation of local alternative structures, such as broken pairs. To test this possibility, we filtered the MD trajectories to keep only those structures (snapshots) where all the hydrogen bonds in the analyzed fragment (inner 27 bp for the longer sequence, inner 19 bp for the shorter one) were intact. In this way, roughly 80% of the snapshots were kept. The resulting twist–temperature slopes, including their errors, were very close to those deduced from the whole trajectories (data not shown). We also carefully verified that there are no other alternative structures, such as kinks, in the MD data. Thus, the thermally induced twist decrease observed in MD is not caused by the presence of broken pairs or other structural defects. Instead, it is the property of the intact simulated double helices themselves.

### Effect of Global Twist Definition

The MD results presented so far refer to the end-to-end twist as defined in the Materials and Methods, a twist angle between two right-handed, orthonormal frames located at the ends of the duplex. The twist angle was computed exactly as the local twist in the 3DNA algorithm,^38^ i.e., as the rotation angle between the end frames measured in the plane whose normal is the mean of the two *z*-axis vectors (mean plane). The end frames, in turn, were projections of the base-pair frames of the end pairs onto the local helical axis. Taking just the end frames into consideration mimics the MT experiments, where the overall rotation between the two ends is controlled, while conformational features in the intervening part are not explicitly included. The 3DNA twist angle definition, moreover, ensures that the end-to-end twist changes are invariant with respect to constant offset rotations of the end frames about their *z*-axes, an offset that is also not known in the MT experiment.^14^

To examine the dependence of our results on the global twist definition, we also determined the global twist as the sum of helical twists (h-twists) over all of the base-pair steps involved. Helical twist definitions used by the 3DNA^38^ and Curves+^39^ conformation analysis programs were tested. They are both based on the axis of the screw transformation mapping one base pair to the next one. However, while 3DNA uses the screw axis directly as the local helical axis, the Curves+ axis is smoothed by using a polynomial weighting function. The temperature change of the sum of 3DNA h-twists (Table S1) underestimates the MT measurement already for dsDNA, as reported previously.^14^ In the case of dsRNA, it is still further from the experimental value than the MD end-to-end twist, being even positive rather than negative in one case (Table S1). The Curves+ data are closer to the MD end-to-end twist values (Table S1), in line with earlier results for dsDNA.^14^ Nevertheless, the twist temperature slopes for the two h-twist definitions are both tightly correlated with the end-to-end twist data (Figure S2). This is understandable: all three definitions ultimately depend on the relative rotations between bases and base pairs, and since the thermal effects are rather small, these dependencies can be linearized, yielding linear relations between the global twist changes computed using any two of the definitions. Taken together, the various global twist definitions examined here consistently indicate a severe underestimation of the dsRNA twist temperature change by MD simulations compared to the measured value.

### Simulations using the TIP3P Water Model

The TIP3P water model^34^ ranks among the most popular water parametrizations used in biomolecular simulations. Here, we tested the model together with the Joung–Cheatham (JC) K^+^/Cl^–^ and Na^+^/Cl^–^ ion parameters^31^ (Materials and Methods), a combination commonly employed in MD simulations of nucleic acids.^35,36^ The simulated systems and the thermally induced twist changes observed are listed in Table S2. In the case of DNA, the simulated change of end-to-end twist is −9 ± 1°/(°C·kbp), further from the experiment than the values deduced using SPC/E water and Dang ions (see above) but still reasonably close to the experimental value. In contrast, the RNA temperature-dependent end-to-end twist data at 150 mM KCl or NaCl and the TIP3P water, for both the 33 and 25 bp oligomers, are scattered, with the slope close to zero and a rather poor linear fit (Table S2 and Figure S3), at variance with the experiment. Increasing the ion concentration to 1 M KCl, we observe a slight increase (rather than a decrease) of twist with temperature, and in the near-ambient temperature range of 25–35 °C, the twist even sharply increases by +5 ± 1°/(°C·kbp) (Table S2 and Figure S4). Thus, employing the TIP3P/JC parameters yields a thermally induced dsRNA twist change entirely at odds with experimental observations. Notice, however, that the JC ions combined with the other water models tested here (SPC/E and TIP4PEw) yield the correct sign of the twist change and are rather close to the SPC/E-Dang simulation. The problem, therefore, is likely at the side of the TIP3P water model, which is known to have a limited capability to reproduce properties of real water and their temperature dependence.^47^ These observations suggest that the TIP3P model cannot be recommended for simulations of temperature-dependent structural changes in nucleic acids, especially dsRNA.

## Discussion

In this work, we reported the changes of twist of nucleic acid double helices with temperature, probed by magnetic tweezer experiments and all-atom MD simulations. We first extended the previous work^14^ to verify that both the current Amber force fields, bsc1 and OL15, yield a dsDNA twist temperature change in quantitative agreement with the MT measurement. We then turned to dsRNA and examined its twist temperature dependence by MT experiment and extensive MD simulations involving different ionic conditions and a range of force fields for dsRNA, water molecules, and ions. The duplex end-to-end twist inferred from the MD data decreased with temperature, qualitatively agreeing with the MT experiments. However, in contrast to the dsDNA case, we found a large discrepancy between the dsRNA twist temperature decrease measured by MT and its prediction by atomistic MD simulations, the latter being less than a third of the experimental value.

To obtain more insight into the microscopic origin of the twist temperature dependence, we complemented our MD data with simulations of a DNA–RNA hybrid oligomer using two different force fields. The MD results including all three duplex variants (DNA, RNA, and hybrid) revealed a tight linear correlation between the twist–temperature slope and the duplex compaction quantified by the helical rise: more compact duplexes with a smaller helical rise also exhibit lower sensitivity of twist to temperature changes. Remarkably, this dependence is also seen for the case of salt-induced compaction of an RNA duplex, where the increase in salt concentration from 150 mM to 1 M KCl results in a more compact structure with a less temperature-sensitive twist (Figure 3 and Table S1). While the linear dependence agrees quantitatively with dsDNA experimental data, it predicts a much weaker twist change with temperature for the dsRNA than experimentally observed due to the much lower dsRNA twist temperature change deduced from MD.

The origin of such a discrepancy is not *a priori* clear. One obvious possibility is some systematic shortcoming of the force fields tested. For instance, the (now obsolete) bsc0 Amber force field^48^ examined in the prior study^14^ underestimated the dsDNA twist temperature decrease by ∼32%. Since the improvement to the current bsc1 and OL15 DNA force fields consists of adjusting the backbone torsional parameters, the possible dsRNA force field problem might be primarily related to the inaccurate description of the backbone. On the other hand, the discrepancy between MD and experiment observed here for dsRNA is much larger than in the case of the bsc0 DNA simulations. Furthermore, the deviation is similar for two entirely different force fields: χOL3 and Shaw. Thus, it appears unlikely that the disagreement with the experiment is caused by the failure of a particular force field. If rooted in a force field bias at all, it might rather be due to general properties of this class of force fields, limited by their functional form and lack of polarization.^35^

We note that a similar study using MT measurements and all-atom MD simulations to probe the dependence of dsDNA twist on both ion concentration and identity observed quantitative agreement for some but also considerable deviations for other ions.^16^ By contrast, the discrepancy between the simulated and experimental twist temperature change observed here is consistently large for all of the ion and water models examined, adding confidence to the robustness of our results. The ability of MD force fields to faithfully reproduce structure, dynamics, and elasticity of DNA and RNA duplexes has been extensively tested.^35,36,49^ For instance, modern dsDNA and dsRNA force fields are able to reproduce, both in sign and in magnitude, even such a subtle effect as the opposite coupling between twist and elongation in DNA and RNA duplexes, where DNA underwinds when stretched, while RNA overwinds.^23,50^ Moreover, all of the simulated duplexes conform to the same linear relation between the helical rise of the duplex and its thermally induced twist change.

These considerations suggest a rather consistent picture, namely, that the ∼30 bp double helices at the microsecond scale sample a certain domain of the conformational space, characterized by a tight correlation between the duplex compaction, quantified by the helical rise, and the sensitivity of its twist to temperature. In contrast to MD, the MT measurements take seconds to minutes and involve kilobase-long duplexes. Thus, they probe the double helix at much larger time and length scales than the MD simulations. There may be structural changes in the RNA duplex taking place at these longer scales, increasing the twist temperature dependence.

Such slow changes are well-known to occur in the DNA duplex. They include the base-pair breathing at the 100 μs scale,^51^ formation of the Hoogsteen pairs at the millisecond scale,^52^ or the exchange between the *a* and *b* states in the, somewhat speculative, two-state model of DNA shape and stiffness.^12^ Moreover, the changes may be cooperative, as in the two-state DNA model where the domain size exceeds 200 bp. Nevertheless, these processes do not seem to significantly affect the DNA twist change with temperature, whose measured value agrees quantitatively with the microsecond-scale MD prediction. By contrast, a large discrepancy is found in the case of the RNA double helix, still awaiting a possible structural explanation. We note that the MT measurements are performed at a low stretching force (∼0.3 pN) that would presumably not preclude the formation of alternative structures. Our simulations predict a change in twist with temperature for the DNA–RNA hybrid intermediate between the values for DNA and RNA. Experimentally testing this prediction would likely provide a plausible route toward understanding why DNA but not RNA twist changes are reproduced by MD simulations.

### Relation to Previous Work

The discrepancy between the thermally induced changes of dsRNA twist measured by MT and inferred from MD simulations that we present here can be contrasted with the results of a recent study of Tian et al.,^25^ where the authors report quantitative agreement between MT measurements and MD simulations. While their MT measurement agrees quantitatively with ours, the MD-derived twist changes differ. We note, however, that Tian et al. did not deduce the temperature-dependent twist directly from the simulated dsRNA structures. Instead, they used the MD data to parametrize a mechanical model and then reported the twist change predicted by the model.

In the model of Tian et al.,^25^ the change of dsRNA helical twist is coupled to major groove deformation. The authors further propose that lowering salt concentration or increasing temperature enlarges the dsRNA major groove, resulting in a twist decrease through the twist–groove coupling. They claim that the mechanism is similar for the two stimuli; the groove is enlarged by phosphate–phosphate electrostatic repulsion or by temperature-dependent entropic force, presumably revealing some universality in dsRNA deformations. They predict a temperature-dependent twist change of −0.012°/(°C·bp), in close agreement with −0.015°/(°C·bp) that they measured using a magnetic tweezer assay.

Tian et al. assume that the free energy *F*(*G*, *T*) associated with the major groove width *G* and temperature *T* takes the form
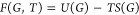
2where the internal energy *U*(*G*) and entropy *S*(*G*) do not depend on temperature, in close analogy with a model proposed earlier.^13^ They infer *F*(*G*, *T*) values from all-atom MD data at 22 and 27 °C, subtract them to deduce the entropy, and fit the entropy by a linear function. The computed twist change critically depends on the slope of the fitting line, *k*_*SG*_ = ∂*S*/∂*G*.

**Figure 4:**
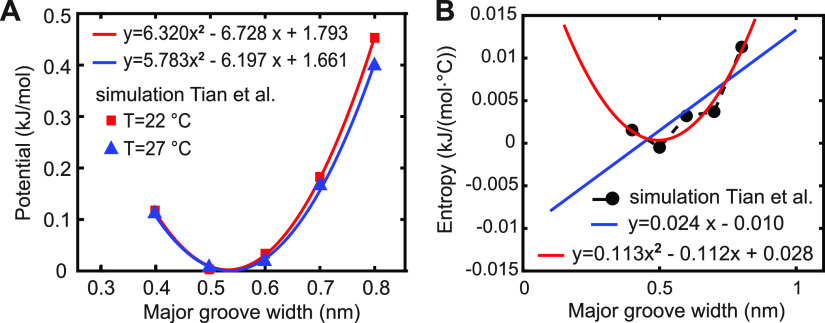
Data points from the work of Tian et al.^25^ together with the fitting functions computed here: (A) free energy, or potential of mean force, and (B) entropy.

However, the model of Tian et al. is in fact consistent with a quadratic entropy function, and the linear fit is inappropriate. To show this, we extracted the *F*(*G*, *T*) data for 22 and 27 °C from their Figure S20 and fitted each of them by a quadratic function (Figure 4A). The fits are very good, and subtracting them yields *S*(*G*) = 0.107*G*^2^ – 0.106*G* + 0.026. To verify this result, we also directly fitted the *S*(*G*) values in their Figure 5B. The quadratic fitting function (Figure 4B, red) is indeed close to the *S*(*G*) obtained by subtraction. Fitting the same data by a linear function (Figure 4B, blue) yields the fitting line identical to that in Tian et al. However, since *S*(*G*) is quadratic, the slope sign and magnitude of the linear fit are arbitrary and depend on the data points selected for the fit.

We now use the data of Tian et al. to actually deduce the temperature-dependent twist change, consistent with their model. Subtracting the fitted minima in Figure 4A yields Δ*G* = 7.02 × 10^–4^ nm/°C. To further verify this result, we write eq 2 as *F*(*G*, *T*) = *F*(*G*, *T*_0_) – (*T* – *T*_0_)*S*(*G*) and insert the fit of *F*(*G*, *T*_0_) from Figure 4A together with the quadratic fit of *S*(*G*) from Figure 4B. We then find the minimum *G*_0_(*T*) of *F*(*G*, *T*) for fixed *T* and take the temperature derivative of *G*_0_(*T*) at *T*_0_. Since the functions are quadratic, the computation is straightforward. Performing the calculation for *T*_0_ = 22 °C and *T*_0_ = 27 °C yields two more estimates of Δ*G*, in addition to the subtracted minima. The three estimated values are close to each other and average to Δ*G* = 7.15 × 10^–4^ nm/°C. Now, Tian et al. express the coupling between *G* and the twist ω by a quadratic elastic energy (their eq 2) with the coefficients inferred from MD (their eq 3). Minimizing the elastic energy for fixed Δ*G*, we find Δω = (− *k*_ω*G*_/*k*_ω_)Δ*G*, and inserting our averaged Δ*G*, we obtain Δω = −1.7 × 10^–3^°/(°C·bp). This is an order of magnitude smaller than the experimental MT value. Thus, when employed more carefully, the model of Tian et al. in fact predicts nearly no change of twist with temperature, challenging the universality of the proposed mechanism. Indeed, while the model of salt-dependent unwinding is physically plausible (change of the P–P repulsion in the major groove and its elastic coupling to the twist), it is not clear why such a mechanism should be in operation for thermally induced changes as well. It is more likely that the structure just thermally expands as a whole in all its parts rather than just the groove expanding and the twist passively following the change through the elastic coupling.

In contrast to the approach of Tian et al., in this work, we do not rely on any mechanical model and just infer the thermally induced twist change directly form the statistical ensemble of MD-generated structures. Among the sequence variants, ionic conditions, NA force fields, and ion and water models tested here, none provides the twist change anywhere near the experimental value. In particular, one of our setups (RNA_25 sequence, 1 M KCl salt, TIP3P water, twist defined as a sum of Curves+ helical twists) exactly corresponds to the protocol of Tian et al. As can be seen in Table S2 and Figure S4, this setup in fact yields a distinct increase of the twist with increasing temperature in the ambient range of 25–35 °C, namely, +5 ± 1°/(°C·kbp). This is at odds not only with the MT experiment but also with the prediction of the (carefully treated) model of Tian et al. shown above.

## Conclusions

We have presented a combination of careful experimental and simulation studies to probe the temperature-dependent twist of the RNA double helix. Both the magnetic tweezer measurements and all-atom MD simulations (with the exception of those employing the inappropriate TIP3P water model) indicate that dsRNA unwinds with a rising temperature. However, the magnitude of the thermally induced twist decrease observed in the simulations is much weaker than that observed in the experiment. While some as yet undiscovered MD force field bias cannot be excluded, we also consider an alternative explanation, namely, that this difference may reflect the existence of transient structures formed in the RNA duplex at time and length scales inaccessible to MD simulations. This would imply, in contrast to DNA, a scale-dependent structural response of the RNA double helix to temperature.

## Supporting Information Available

RNA sequence used in the MT experiment and additional tables and figures showing experimental and simulated data (PDF)
