# Supplementary material for: Temperature-Dependent Twist of Double-Stranded RNA Probed by Magnetic Tweezer Experiments and Molecular Dynamics Simulations
Source: J Phys Chem B. 2024 Jan 10;128(3):664–75. doi: 10.1021/acs.jpcb.3c06280 (PMC10823466; doi:10.1021/acs.jpcb.3c06280)
Supplement: Supplementary file 1 — jp3c06280_si_001.pdf [file jp3c06280_si_001.pdf]

SUPPORTING INFORMATION

Temperature-Dependent Twist of Double-Stranded RNA Probed by Magnetic Tweezer Experiments and Molecular Dynamics Simulations

Hana Dohnalová†, Mona Seifert†, Eva Matoušková, Misha Klein, Flávia S. Papini, Jan Lipfert, David Dulin*, Filip Lankaš*

† Equal contribution

* Corresponding authors: filip.lankas@vscht.cz, d.dulin@vu.nl

Supplementary Methods

Sequence of the stem between the biotin- and the digoxigenin-labelled handles:

AAAACCGTATGACGCTGGAAGCGATTCGTGTCACCCAGTCGGACCGCGACCTGTTTAAGCATCTGATCAG

CGAAGCCACCAACTACGTGGCGGCGGACTACATGCGTCACGCCAACGAGCGCCGTGTCCATCTCGACAAA

GCCCTGGAGTTTCGTCGCGAGCTACATACTTCGCGTCAGCAACTGGCGGCTGAGCAGTACAAACACGTCG

ATATGGCGCGTGAGCTGGCAGAGCACAACGGTGCCGAAGGCGATCTGGAAGCGGATTATCAGGCGGCCAG

TGATCACCTGAACCTGGTGCAAACCGCACTGCGTCAGCAGGAGAAGATCGAACGCTACGAAGCGGATCTC

GATGAGCTGCAGATCCGTCTGGAAGAGCAAAATGAAGTGGTGGCAGAAGCCATCGAACGCCAGCAAGAGA

ATGAGGCTCGTGCGGAAGCTGCCGAACTGGAAGTGGACGAGCTGAAAAGCCAGCTTGCTGACTACCAGCA

GGCGCTGGACGTCCAGCAAACGCGCGCGATCCAGTATAACCAGGCGATTGCTGCGCTTAATCGTGCCAAA

GAACTGTGCCATCTGCCGGACTTAACCGCCGACTGCGCCGCCGAATGGCTGGAAACCTTCCAGGCGAAAG

AGCTGGAAGCGACTGAAAAAATGCTCTCTCTTGAGCAGAAAATGAGCATGGCGCAAACCGCGCACAGCCA

GTTTGAGCAGGCTTATCAGCTGGTGGTGGCAATCAACGGCCCACTGGCGCGTAACGAGGCGTGGGATGTC

GCTCGCGAACTATTGCGCGAAGGGGTCGATCAGCGTCACCTGGCAGAGCAGGTTCAGCCGCTGCGGATGC

GATTAAGCGAACTGGAACAGCGTCTACGCGAGCAGCAAGAAGCTGAGCGTCTGCTGGCAGATTTCTGCAA

ACGTCAGGGCAAGAATTTTGATATCGACGAACTGGAAGCCCTGCATCAGGAACTGGAAGCACGCATTGCC

TCTCTTTCCGATAGCGTGTCTAACGCCCGTGAAGAGCGCATGGCACTGCGCCAGGAGCAGGAACAGCTGC

AGTCTCGCATTCAGAGTTTGATGCAGCGTGCGCCGGTTTGGCTGGCAGCGCAAAACAGTCTCAACCAGTT

GAGCGAACAGTGCGGCGAAGAGTTTACCTCCAGCCAGGACGTCACCGAATATCTGCAACAGTTGCTGGAG

CGTGAGCGAGAGGCGATTGTTGAACGCGATGAAGTGGGCGCGCGCAAAAACGCCGTCGATGAAGAGATCG

AACGTTTAAGCCAGCCTGGCGGCTCTGAAGATCAGCGTCTGAACGCGCTGGCGGAGCGTTTTGGTGGTGT

GCTGCTGTCAGAAATTTATGACGACGTTAGCCTGGAAGATGCGCCGTACTTCTCAGCGCTGTATGGCCCG

TCACGCCACGCCATCGTGGTGCCAGATCTGTCACAGGTAACTGAACACCTGGAAGGCTTGACCGATTGCC

CGGAAGATCTCTATCTGATCGAAGGAGATCCGCAGTCATTCGATGACAGCGTGTTCAGCGTTGATGAGCT

GGAAAAAGCGGTAGTGGTGAAAATCGCCGATCGTCAGTGGCGTTATTCACGTTTCCCGGAAGTGCCGCTG

TTTGGTCGTGCTGCGCGTGAAAGCCGTATTGAAAGCCTCCATGCCGAGCGTGAAGTGCTTTCCGAACGCT

TCGCCACGCTCTCCTTTGATGTACAGAAAACTCAGCGTCTGCATCAGGCGTTCAGCCGCTTTATCGGCAG

TCATCTGGCGGTTGCGTTTGAGTCTGACCCGGAAGCAGAAATCCGTCAACTGAACAGCCGTCGCGTCGAA

CTGGAGCGGGCGTTAAGTAATCATGAAAATGATAACCAGCAGCAGCGTATTCAGTTTGAGCAGGCGAAAG

AGGGCGTTACGGCGCTGAACCGCATTCTGCCGCGTCTCAACCTGTTGGCTGATGACAGCCTGGCGGATCG

CGTCGATGAAATCCGCGAACGTCTGGATGAAGCCCAGGAAGCCGCGCGTTTTGTTCAGCAGTTTGGCAAT

CAACTGGCGAAACTGGAACCGATTGTTTCGGTATTGCAGAGCGACCCGGAACAGTTCGAACAGTTAAAAG

AAGATTACGCGTACTCTCAGCAGATGCAGCGCGATGCCCGTCAGCAGGCGTTTGCCCTGACGGAAGTGGT

GCAGCGTCGTGCGCACTTTAGCTATTCTGACTCGGCAGAAATGCTTAGCGGTAACAGCGATCTCAACGAA

AAACTGCGTGAACGTCTGGAACAGGCGGAAGCGGAGCGTACCCGCGCTCGCGAAGCGTTGCGCGGTCACG

CAGCGCAGTTGAGTCAGTACAACCAGGTGCTGGCTTCGCTGAAAAGTTCTTACGACACCAAAAAAGAGCT

ACTCAACGATCTGCAACGTGAATTGCAGGATATCGGCGTGCGTGCTGATAGCGGGGCAGAAGAGCGGGCG

CGTATTCGCCGTGACGAGCTGCATGCGCAACTGAGCAATAACCGTTCACGCCGCAATCAACTGGAAAAAG

CGCTTACCTTCTGCGAAGCGGAGATGGACAACCTGACCCGCAAACTGCGCAAGCTGGAGCGGGATTACTT

TGAGATGCGCGAGCAGGTAGTGACCGCCAAAGCGGGCTGGTGTGCGGTGATGCGCATGGTGAAAGATAAC

GGCGTTGAGCGCCGCTTACACCGTCGTGAGCTGGCTTATCTCTCCGCTGATGATTTGCGTTCCATGTCGG

ATAAGGCGTTAGGTGCGCTGCGTCTGGCGGTGGCGGATAACGAACATCTGCGCGACGTGCTGCGCATGTC

GGAAGATCCGAAACGTCCGG

Table S1. Change of twist with temperature obtained from molecular dynamics (MD) simulations. The numbers in italics are square correlation coefficients indicating goodness of the linear fit. The errors of the h-rise are very small and are not indicated.

sequence	Salt conc. (M)	NA force field water, ions	ΔTw/ΔT[a] (deg·°C^{-1}·kbp^{-1})	ΔhTw/ΔT[b] (deg·°C^{-1}·kbp^{-1})	ΔhTw/ΔT[c] (deg·°C^{-1}·kbp^{-1})	h-rise (Å)
DNA_33	0.15	OL15<br>SPC/E Dang	-10.11 ± 0.32<br>*0.98*	-9.81 ± 0.30<br>*0.98*	-6.85 ± 0.39<br>*0.96*	3.23
DNA_33	0.15	bsc1<br>SPC/E Dang	-11.73 ± 0.09<br>*0.99*	-11.77 ± 0.20<br>*0.99*	-8.20 ± 0.10<br>*0.97*	3.23
DNA-RNA_33	0.15	OL15/Chiol3<br>SPC/E Dang	-6.69 ± 0.08<br>*0.97*	-6.84 ± 0.20<br>*0.99*	-3.66 ± 0.40<br>*0.88*	2.92
DNA-RNA_33	0.15	bsc1/Chiol3<br>SPC/E Dang	-5.41 ± 0.62<br>*0.89*	-4.65 ± 0.92<br>*0.88*	-1.85 ± 0.44<br>*0.43*	2.94
RNA_33	0.15	Chiol3<br>SPC/E Dang	-4.47 ± 0.22<br>*0.97*	-4.11 ± 0.14<br>*0.97*	-1.52 ± 0.21<br>*0.96*	2.66
RNA_33	0.15	Shaw<br>TIP4P-D C22	-3.97 ± 0.16<br>*0.91*	-3.72 ± 0.15<br>*0.91*	-0.75 ± 0.11<br>*0.27*	2.64
RNA_33	0.15	Chiol3<br>SPC/E JC	-2.99 ± 1.14<br>*0.76*	-2.02 ± 1.11<br>*0.45*	-0.05 ± 1.21<br>*0.00*	2.56
RNA_33	0.15	Chiol3<br>TIP4PEw JC	-2.27 ± 0.60<br>*0.98*	-1.99 ± 0.41<br>*0.98*	-0.75 ± 0.27<br>*0.58*	2.57
RNA_25	0.15	Chiol3<br>SPC/E Dang	-4.08 ± 0.93<br>*0.93*	-3.95 ± 0.95<br>*0.91*	-0.89 ± 0.91<br>*0.23*	2.65
RNA_33	1	Chiol3<br>SPC/E Dang	-2.93 ± 0.09<br>*0.96*	-2.37 ± 0.11<br>*0.95*	-0.15 ± 0.10<br>*0.09*	2.61
RNA_25	1	Chiol3<br>SPC/E Dang	-3.28 ± 0.50<br>*1.00*	-3.17 ± 0.54<br>*1.00*	-0.43 ± 0.54<br>*0.47*	2.61
RNA_33[d]	0.15	Chiol3<br>SPC/E Dang	-3.09 ± 0.71<br>*0.96*	-2.60 ± 0.69<br>*0.94*	0.05 ± 0.73<br>*0.01*	2.64
RNA_25[d]	0.15	Chiol3<br>SPC/E Dang	-3.40 ± 1.03<br>*0.91*	-3.19 ± 0.97<br>*0.88*	-0.72 ± 0.92<br>*0.41*	2.65

[a] MD values obtained using the end-to-end twist.

[b] MD values obtained using the sum of Curves+ helical twists.

[c] MD values obtained using the sum of 3DNA helical twists.

[d] MD simulations with NaCl.

Table S2. Change of twist with temperature obtained from molecular dynamics (MD) simulations using the TIP3P water model. The numbers in italics are square correlation coefficients indicating goodness of the linear fit.

Sequence	Salt conc. (M)	NA force field	ΔTw/ΔT[a] (deg·°C^{-1}·kbp^{-1})	ΔhTw/ΔT[b] (deg·°C^{-1}·kbp^{-1})	ΔhTw/ΔT[c] (deg·°C^{-1}·kbp^{-1})	H-Rise (Å)
DNA_33	0.15	OL15	-8.95 ± 0.84<br>*0.98*	-8.67 ± 0.84<br>*0.98*	-5.58 ± 0.75<br>*0.96*	3.23 ± 0.00
RNA_33	0.15	Chiol3	-1.22 ± 0.78<br>*0.70*	-0.60 ± 0.90<br>*0.29*	0.78 ± 0.30<br>*0.24*	2.65 ± 0.00
RNA_25	0.15	Chiol3	0.32 ± 0.00<br>*0.01*	0.89 ± 0.02<br>*0.07*	3.89 ± 0.31<br>*0.72*	2.65 ± 0.01
RNA_25	1	Chiol3	1.17 ± 0.29<br>*0.45*	1.49 ± 0.23<br>*0.57*	3.83 ± 0.05<br>*0.89*	2.56 ± 0.01
RNA_25[d]	1	Chiol3	5.03 ± 1.28<br>*0.94*	4.96 ± 1.02<br>*0.94*	6.46 ± 0.17<br>*0.91*	2.56 ± 0.01
RNA_33[e]	0.15	Chiol3	1.56 ± 1.34<br>*0.40*	2.31 ± 1.18<br>*0.63*	4.89 ± 1.20<br>*0.88*	2.69 ± 0.01

[a] MD values obtained using the end-to-end twist.

[b] MD values obtained using the sum of Curves+ helical twists.

[c] MD values obtained using the sum of 3DNA helical twists.

[d] Changes of twist between 25 and 35 °C.

[e] MD simulations with NaCl.

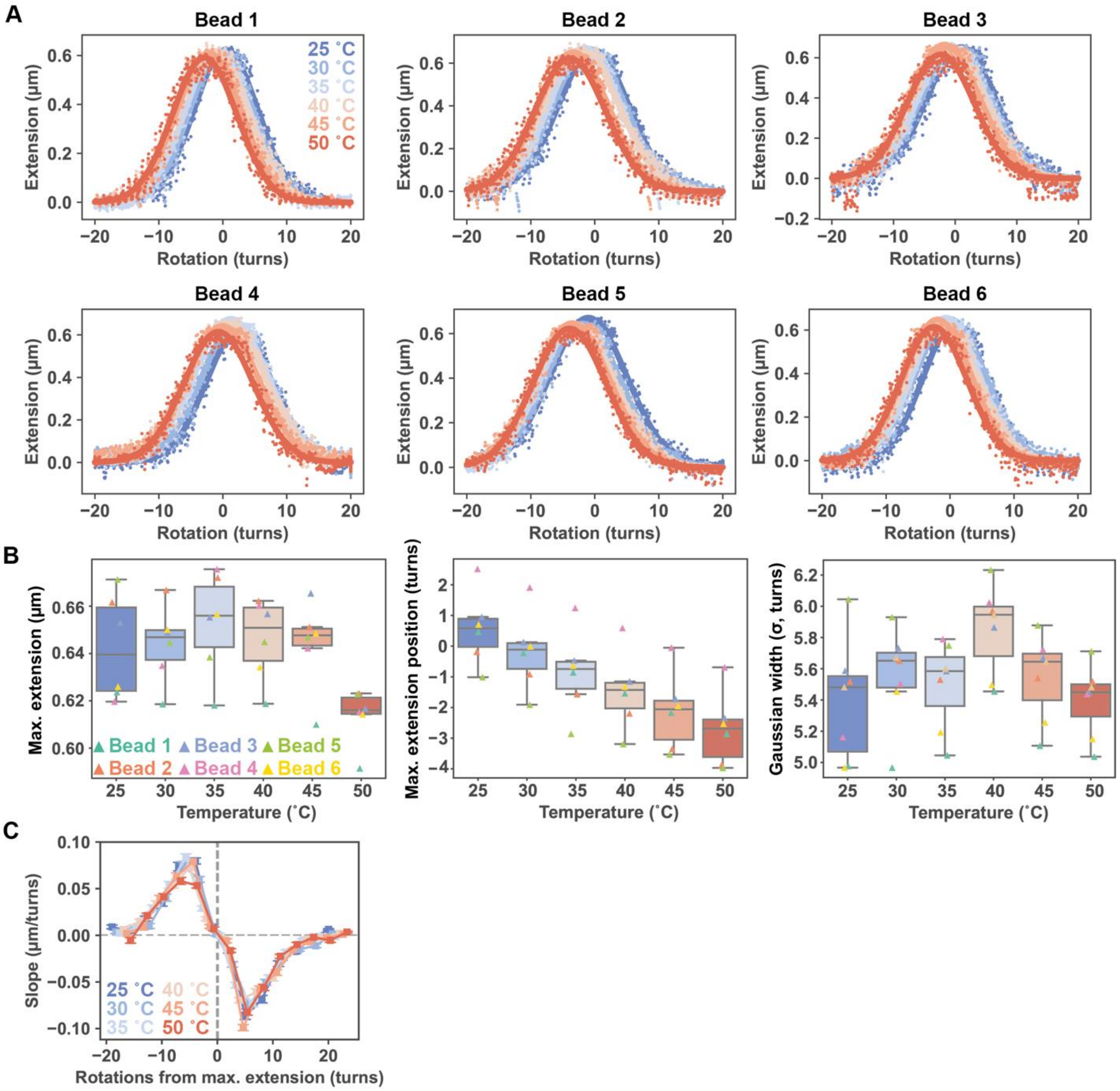

Figure S1. Temperature-dependence of the rotation-extension curves for individual coilable dsRNA tethers. (A) Individual rotation-extension curves (dots) for temperatures varying from 25°C to 50°C (color code indicated in the "Bead 1" panel). Each dataset is shown together with its Gaussian fit (solid line) in the same color. **(B)** Gaussian fit parameters, with the maximum extension (left), the maximum extension position (center) and the width (standard deviation, right) obtained from the fits in (A). **(C)** Local slope of the rotation-extension curves from datapoints shown as dots in (A). All data at a given temperature were aggregated into one combined dataset and the slope of the regression line was found for all datapoints within non-overlapping 2-turn windows. Error bars represent the standard deviation in the fitted slopes. Datasets have been aligned by their respective peak positions to enable a direct comparison across different beads as well as temperatures.

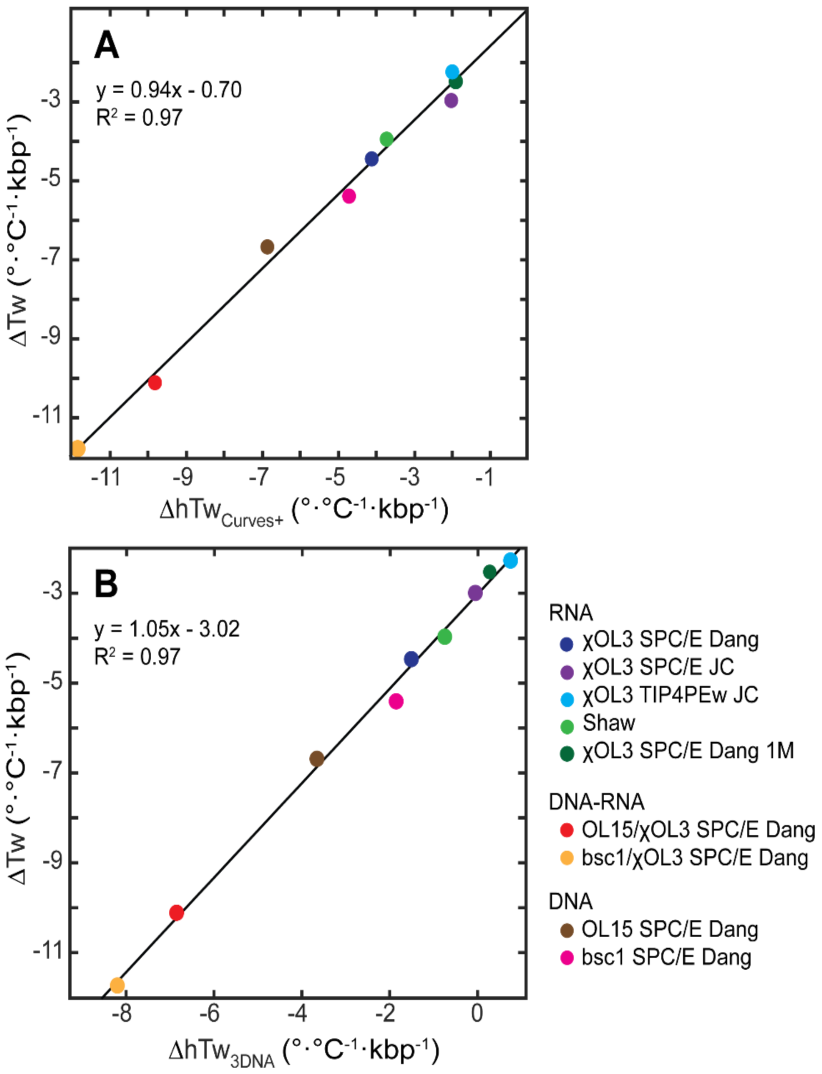

Figure S2. Relations between twist-temperature slopes computed using various definitions of the oligomer global twist. (A) End-to-end twist and sum of helical twists (h-twists) extracted from the Curves+ output, **(B)** end-to-end twist and sum of h-twists computed as in 3DNA. The equation of the fitting line and the square correlation coefficient are also shown. The colour coding indicated holds for both panels.

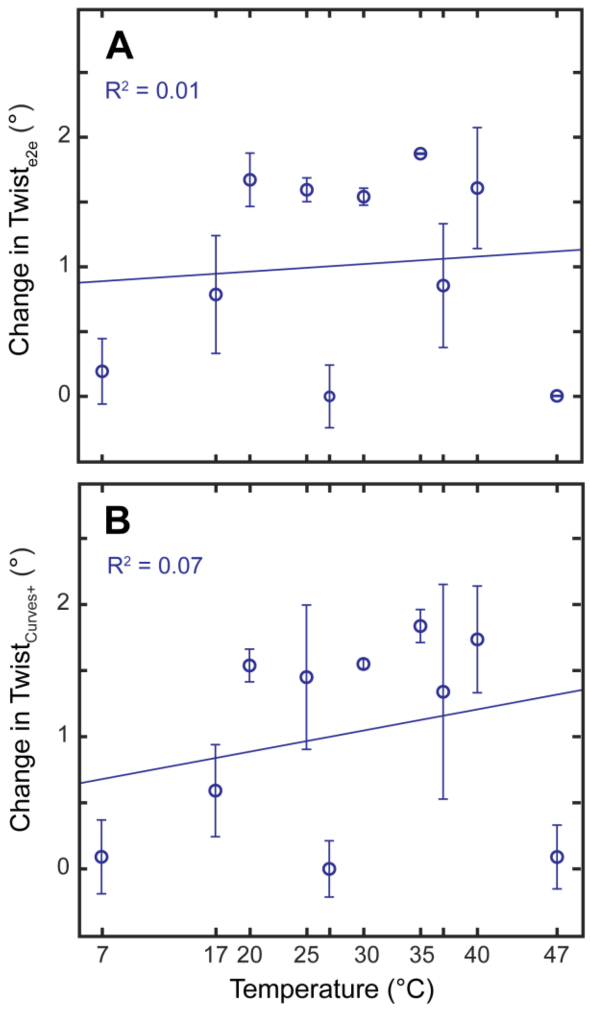

Figure S3. Simulated temperature-dependent change in twist for the RNA_25 sequence at 150 mM KCl using the TIP3P water model. The values of end-to-end twist (A) as well as the sum of helical twists computed by Curves+ (B) are scattered and yield a poor linear fit with a slope close to zero.

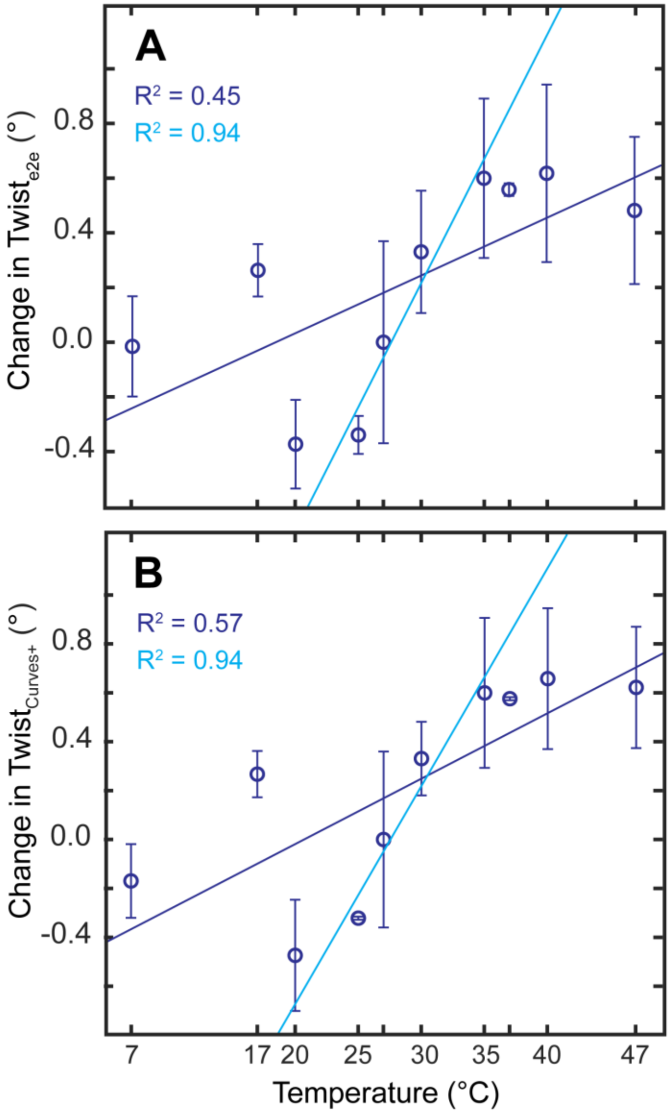

Figure S4. Simulated temperature-dependent change in twist for the RNA_25 sequence at 1 M KCl using the TIP3P water model. The values of end-to-end twist (A) as well as the sum of helical twists computed by Curves+ (B) exhibit a slight increase over the whole temperature range of 7 to 47 °C, as indicated by the line fitting all the data points (dark blue line). However, if the fit is performed over the data points in the near-ambient range of 25-35 °C only (cyan line), a sharp increase of the twist with temperature is observed.
